# Infuence of co-morbidity in the prognosis of politrauma in geriatric patients

**DOI:** 10.1186/1471-2318-11-S1-A12

**Published:** 2011-08-24

**Authors:** F Famà, L M Murabito, A Beccaria, F Cucinotta, A Caruso, C D Foti, M A Gioffrè Florio

**Affiliations:** 1 Unità Operativa Complessa di Medicina e Chirurgia d’Accettazione e d’Urgenza con Osservazione Breve - Azienda Ospedaliera Universitaria “G.Martino” di Messina, Italy

## Background

In geriatric patients co-morbidity, i.e. the presence of several diseases simultaneously, is frequent, and exerts a decisive influence on elderly patients’ heading progressively towards complete loss of autonomy and exposure to very high risk injury. In the case of trauma and even more of polytrauma, the high level of comorbidity and drug therapies in the elderly, influence the outcome and the approach of medical treatment with important implications for both diagnostic and therapeutic plans. The increasing number of drugs (polypharmacy) required with increasing concomitant diseases (cardiovascular, neurological, musculoskeletal, etc.) stretch to influence the therapeutic response, with side effects that sometimes complicate multiple trauma, making therapeutic strategies particularly complex and difficult to manage.

## Materials and methods

**Figure 1:**
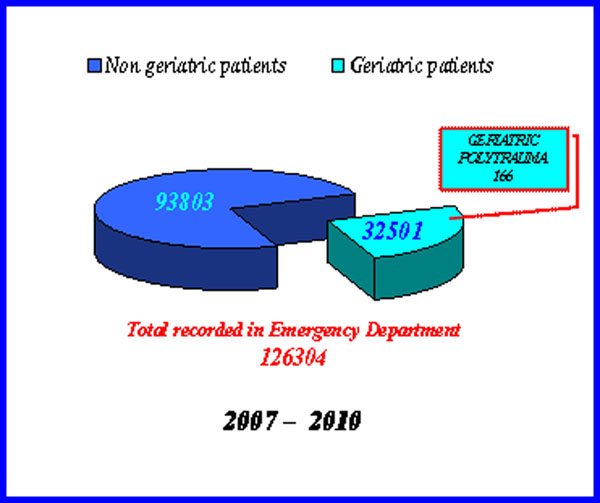


In the period January 2007 - October 2010 we recorded in our emergency department, an influx of geriatric patients (≥65 years) amounting to 32501 (25.73%), amongst these 166 had multiple traumas (Fig.1). The clinical conditions in patients’ records, quickly identified with the color code (White-Green-Yellow-Red), are framed and evaluated in accordance with the Card of the Trauma Complex in use in our Complex Operative Unit of Emergency Medicine Surgery Acceptance with OB. The Probability of survival (Ps) is calculated by the method TRISS (Trauma and Injury Severity Score), or with the ISS (Injury Severity Score).

## Results

**Figure 2:**
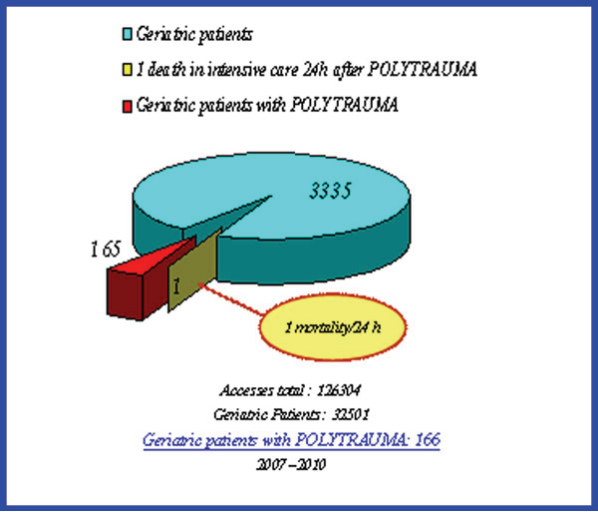


During the considered period, we observed a constant number of cases of multiple trauma in geriatric patients, with a high number of females above the age group between 75 and 84 years. Over 60% of patients assessed by the ISS, had a score >15 (V-class seriousness of severe injuries) and just over 30%, a score <15 (class I to IV severity). All patients were hospitalized and we recorded only one death in intensive care 24hr after trauma (Fig.2).

## Conclusions

The patients with politrauma, had often impairment of vital functions, so the presence of comorbidity in geriatric patients is a state that can increase the risk of mortality and permanent disability. The frailties of the geriatric patient due to the presence of comorbidity makes it more difficult to handle and manage them in emergency conditions. Medical treatment is aimed at managing comorbidities. It is of note that, in our experience, the only death in polytrauma in 24 hours, from 2007 to 2010, was recorded in a geriatric patient.
